# Phasing coherently illuminated nanocrystals bounded by partial unit cells

**DOI:** 10.1098/rstb.2013.0331

**Published:** 2014-07-17

**Authors:** Richard A. Kirian, Richard J. Bean, Kenneth R. Beyerlein, Oleksandr M. Yefanov, Thomas A. White, Anton Barty, Henry N. Chapman

**Affiliations:** Center for Free-Electron Laser Science, Deutsches Elektronen-Synchrotron, Notkestrasse 85, Hamburg 22607, Germany

**Keywords:** protein crystallography, coherent diffractive imaging, free-electron laser

## Abstract

With the use of highly coherent femtosecond X-ray pulses from a free-electron laser, it is possible to record protein nanocrystal diffraction patterns with far more information than is present in conventional crystallographic diffraction data. It has been suggested that diffraction phases may be retrieved from such data via iterative algorithms, without the use of *a priori* information and without restrictions on resolution. Here, we investigate the extension of this approach to nanocrystals with edge terminations that produce partial unit cells, and hence cannot be described by a common repeating unit cell. In this situation, the phase problem described in previous work must be reformulated. We demonstrate an approximate solution to this phase problem for crystals with random edge terminations.

## 1. Introduction

The availability of brief, intense and coherent X-ray pulses produced by X-ray free-electron lasers (XFELs) has created the potential for major advancements in macromolecular crystallography [1,2]. Serial femtosecond crystallography (SFX) [3] is among the most successful new paradigms to emerge, which involves directing a stream of randomly oriented protein crystals across the focus of the XFEL beam. SFX data often consist of hundreds of thousands of diffraction patterns, which can be collected in a matter of hours at current pulse-repetition rates. These diffraction patterns are largely free from radiation damage [4] because the timescales of the relevant damage mechanisms are longer than the exposure time [5,6]. This creates, for example, new possibilities to study irreversible dynamic systems [7], radiation-sensitive targets [8] and macromolecules that only form small (approx. 1 µm) crystals and therefore pose difficulties for conventional synchrotron facilities [9].

As with conventional crystallography, the well-known ‘phase problem’ must be solved in order to reconstruct a real-space electron density map from the measured SFX intensities. Following data processing and reduction [10], some conventional macromolecular crystallography techniques can be readily applied to SFX data. For example, initial phase estimates have been obtained from SFX intensities by molecular replacement, which is effective when a similar known structure is available. On the other hand, *ab initio* phasing methods such as (multiple) isomorphous replacement or multi/single-wavelength anomalous dispersion are under development for XFEL sources [11].

Although a range of effective crystallographic phasing methods exist, there remains a general need for *ab initio* methods that apply to large molecules at medium or low resolution and do not require similar known structures, isomorphous derivatives or resonant diffraction [12]. It was recently suggested that coherently illuminated nanocrystals can provide sufficient information for *ab initio* phasing [13]. The method likely requires diffraction patterns from hundreds of thousands of individual sub-micrometre crystals, which may be obtained via the SFX technique. In the following sections, we consider complications with this approach that arise when the crystals considered in the ensemble do not terminate exactly at the nominal unit cell boundaries, a considerable problem that was not addressed in previous work. Whereas crystal size and shape distributions are of relatively little consequence, we show that the presence of molecular vacancies at the crystal boundaries obscure the notion of the crystal unit cell and necessitates a reformulation of the problem. In this manuscript, we suggest an approximate means of solving this problem, which we demonstrate through simulations.

## 2. Phasing methods for coherently illuminated nanocrystals

Among the most striking observations made during the first SFX experiments in 2009 were the distinct intensity distributions observed around Bragg peaks, attributed to the finite lattice of the crystal. These so-called finite-lattice transforms arise when the coherence length of the illumination spans the full width of a finite crystal [3]. The presence of finite-lattice transforms suggests the application of a key idea by Sayre, who suggested that diffraction patterns can be phased *ab initio* if the intensities are sampled *between* Bragg reflections, according to Shannon's sampling theorem [14]. Attempts to extract continuous intensity maps (‘molecular transforms’) without a highly coherent source were first made by Perutz and others near the time of Sayre's paper [15], though this approach, which requires physical modification of the crystal, has not seen significant use.

The problem of phasing diffraction data from coherently illuminated nanocrystals with varying size and shape has been considered recently, in light of the possibility to collect relevant diffraction data from XFEL sources [13,16–20]. These investigations, which aim to determine the contents of the crystal's unit cell, differ from related work aimed at mesoscopic observations such as strain fields [21–23]. They also differ from previous work that has focused on careful analysis of diffraction data from individual crystals with high signal-to-noise ratio (e.g. [24,25]).

The ensembles of crystals considered by Spence *et al.* were assumed to be constructed by repeated translations of a common unit cell electron density. Under that assumption, the averaged diffracted intensity *I*(***q***) of many crystals is proportional to the product of a squared unit-cell transform *|F*(***q***)*|*^2^, and a mean squared finite-lattice transform 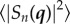
2.1
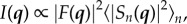
where ***q*** is the momentum transfer vector and *n* denotes the pattern number. As 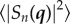 is a periodic function, it may be determined by averaging the diffraction intensity profiles within all Wigner–Seitz cells, which ‘averages out’ the unit-cell transform and effectively decouples the two terms in equation (2.1). Dividing equation (2.1) by the finite lattice transform reveals the transform of the common physical unit cell that repeats throughout the entire crystal, which may be phased via numerous iterative procedures employed in coherent diffractive imaging [26].

Challenges associated with noise, particularly in regions far from the Bragg condition where measured intensity is likely to be low, have been considered [19]. Possible solutions include noise filtering [13], selective sampling [16] or iterative algorithms that directly use only Bragg peak intensities and their associated intensity gradients [18]. Internal crystal disorder has also been considered, in which case the incorporation of partial coherence models into phasing algorithms can effectively improve resolution [17].

Whereas previous work has assumed a common molecular arrangement for every unit cell, a different situation may arise for space groups other than P1, in which a nominal unit cell contains multiple symmetry-related molecules. It is likely that many such crystals do not assemble in whole-unit-cell increments; some of the symmetry-related molecules that make up a nominal unit cell may be absent near the boundary of the crystal. The phasing methods considered previously do not apply directly to this situation because a unit cell that is common throughout the entire crystal does not exist, and hence equation (2.1) cannot be applied. Below, we show that in some cases, the data reduction scheme proposed by Spence *et al.* may still be used to recover the electron density of the asymmetric unit that composes the crystal, provided that the phasing algorithm is modified appropriately.

## 3. Electron densities of finite crystals

Consider a finite crystal in which the nominal unit cell contains one molecule and one symmetry-related copy. We define the electron density of the molecular asymmetric unit as *A*(***r***), and its symmetry mate as *B*(***r***). The symmetry mate is related to the asymmetric unit by a rotation **R** and a translation ***t***3.1
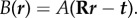


In order to define a complete finite crystal, we define two finite sub-lattices *α*(***r***) and *β*(***r***) for the asymmetric unit and its symmetry mate, respectively. We may express the sub-lattices as3.2
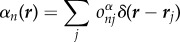
and3.3
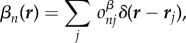
which differ only by the molecular occupancies 
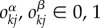
 for each lattice point ***r****_j_*. We can build the electron density of the *n*th crystal by convolving the sub-lattices with their respective molecular electron densities3.4
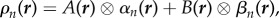
where ⊗ denotes a convolution.

In this framework, we note that one can choose a different *nominal* unit cell by translating *B*(***r***) by integer multiples of the three crystal lattice vectors ***a***, ***b*** and ***c***. In other words, we are free to replace *B*(***r***) with *B*(***r*** + ***d***), where ***d*** = *n_a_**a*** + *n_b_**b*** + *n_c_**c*** and *n_a_* are integers. This has no consequence at the Bragg condition since translations ***d*** produce integer multiples of 2*π* phase shifts at those points in reciprocal space. However, when considering intensities from finite crystals that do not lie at the Bragg condition, care must be taken to make corresponding changes to the occupancies 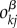 whenever the nominal unit cell (i.e. the vector ***d***) is re-defined. The *physical* unit cell of the crystal, if one exists, should be distinguished from this nominal unit cell. A physical unit cell can be defined only if one can choose a vector ***d*** such that 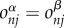 for all *j*. In other words, a physical unit cell can be assigned to a finite crystal *only* if the crystal can be assembled purely from a common unit cell that spans the entire volume of the crystal.

## 4. The average finite-crystal diffraction intensity

Under the Born approximation, the far-field diffraction amplitude is proportional to the Fourier transform 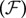 of *ρ_n_*(*r*)4.1
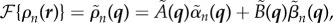
where4.2
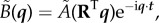
4.3
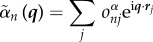
4.4
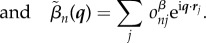
The diffracted intensity is proportional to4.5
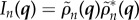

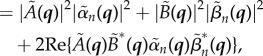
and the average diffracted intensity, arising from a distribution of many crystals with variable occupancies 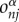 and 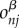, is4.7
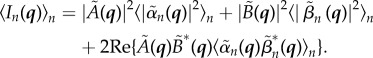
In the special case 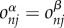, in which the crystal has an unambiguous physical unit cell, equation (4.7) may be simplified to the form described in previous work4.8
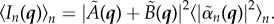


A more realistic model is one in which the crystals are fully occupied up to some boundary, outside of which the occupancies 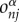 and 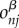 are equal to zero. The statistical properties of 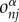 and 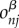, in particular the correlations between them, should be in correspondence with the nature of the crystal growth kinetics, which may vary considerably between different crystal types. In the following sections, we will only consider a simple case in which the free energy associated with the adhesion of a molecule to the crystal surface is identical for all surface molecules.

## 5. Finite one-dimensional crystals

**Figure 1.**
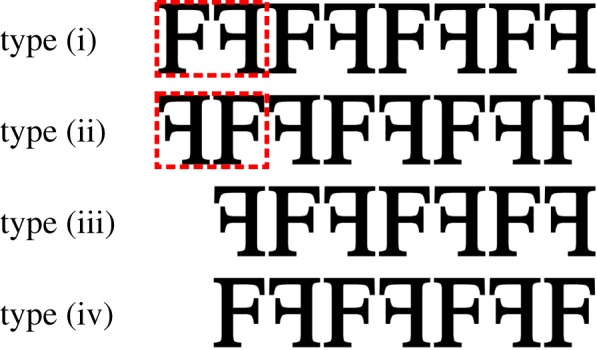
Illustration of four unique types of truncated one-dimensional crystals with two molecules per unit cell. The two unit cell conventions are indicated by the dashed red boxes. Crystal type (ii) is generated from type (i) by switching the first molecule with the second. Types (iii) and (iv) are generated from types (i) and (ii), respectively, by removing the first molecule. (Online version in colour.)

For illustrative purposes, consider a one-dimensional crystal with two molecules per unit cell, and a unit cell length of *a*. We will consider an equal mixture of crystals having all possible edge terminations, including those that leave incomplete unit cells at the crystal boundaries. Schematically, the four possible crystal types are shown in figure 1.

We can see that types (i) and (ii) are ideal crystals that differ only by their physical unit cell; we may produce type (ii) from type (i) by re-defining the symmetry mate as5.1
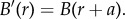


For brevity, we drop the explicit ***q*** dependence and write the average diffraction intensity from these two types as5.2
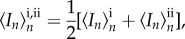
where 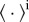 represents the average over crystals with unit cell (i), and 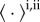 represents the average over crystals with both unit cell definitions (i) and (ii). Thus, if the method prescribed by Spence *et al.* is applied to the averaged intensity arising from these two types of ideal crystals, the average over the two *different* unit-cell transforms will be obtained.

Types (iii) and (iv) do not have a well-defined unit cell. They can be understood as follows. Firstly, we see that type (iii) may be formed by removing the first molecule from type (i). Mathematically, this operation may be performed by making the change^1^ 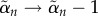 in equation (4.7), which produces the average diffraction intensity5.3
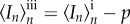
5.4
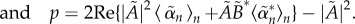
Similarly, the average crystal type (iv) can be produced from type (ii) by making the change 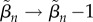
5.5
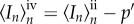
5.6
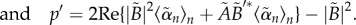


Finally, the combined average over all four crystal types is5.7
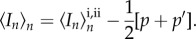
We can see that the average crystal diffraction contains a prominent term equal to the average over two possible unit cell definitions. The additional terms *p* and *p*′ arise from the interference of idealized crystals with a single ‘missing’ molecule at the boundary of the crystal. In the following sections, we consider the feasibility of phasing the averaged diffraction intensities from an ensemble of randomly terminated crystals based on the conjecture that these interference terms may be neglected.

## 6. Finite two-dimensional crystals

**Figure 2.**
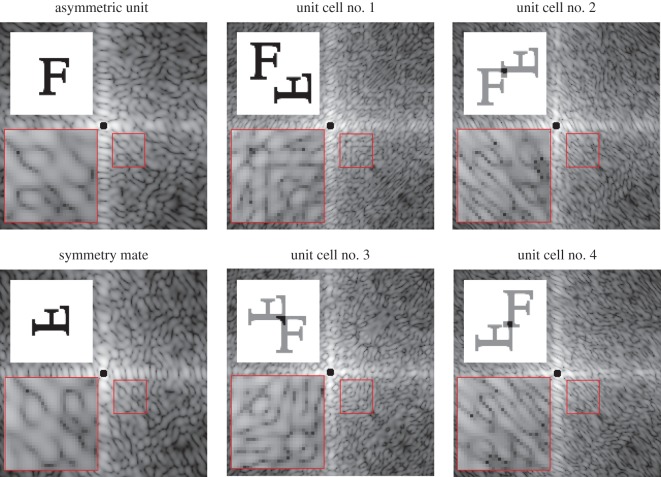
The real-space density (upper insets) and diffraction from the asymmetric unit, symmetry mate, and four compact nominal unit cell definitions. Lower insets show enlarged regions indicated by red boxes. (Online version in colour.)

As the enumeration of two-dimensional crystal types is considerably more complicated than for one-dimensional crystals, we will proceed with simulations. For simplicity, we will assume a square lattice defined by the vectors ***a*** = [*a*, 0] and ***b*** = [0, *a*]. We consider the plane group *cm* [27] with the symmetry mate defined by the rotation (a reflection in two-dimensional projection) and translation6.1
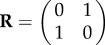
and6.2
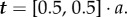
The real-space densities and diffraction intensities from the asymmetric unit, symmetry mate and four different nominal unit cells are shown in figure 2.

**Figure 3.**
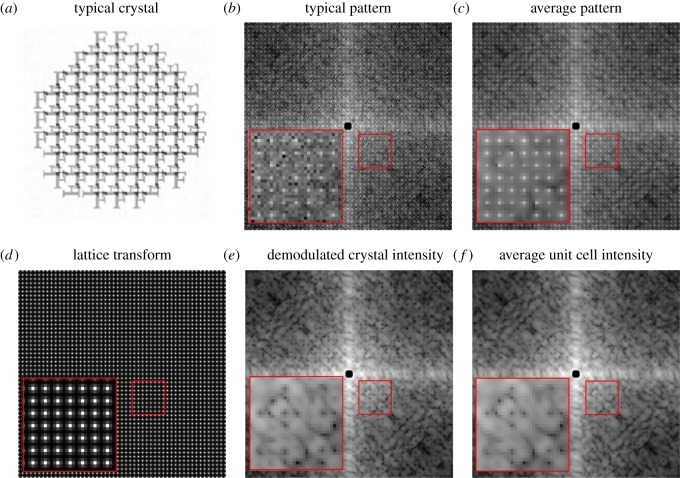
(*a*) A typical crystal, (*b*) typical diffraction pattern and (*c*) the average over 500 diffraction patterns. (*d*) The average Wigner–Setz cell is divided to obtain (*e*) the demodulated crystal intensity map which may be compared with (*f*) the incoherent average over the four compact nominal unit cell definitions shown in figure 2. Lower insets show enlarged regions indicated by red boxes. (Online version in colour.)

We generated 500 diffraction patterns from random circle-shaped crystals with a flat distribution of radii equal to 4*a* ± 0.5*a*. We ensured randomized edge terminations by randomly shifting the origin of each crystal lattice and retained only the lattice points for molecules that fell within the circular boundary with a fixed origin. The intensities were sampled such that three measurements lie between adjacent Bragg reflections, and the maximum Miller index extended out to *h*, *k* = 25. We added random, uniform jitter to the scattering vectors corresponding to each two-dimensional pixel. The addition of jitter emulates a physical detector, which averages over a finite solid angle, and avoids aliasing that would otherwise result from the fine lattice fringes and comparably coarse pixel spacings. We then divided the average crystal intensity by its average Wigner–Seitz cell to obtain what we refer to as the demodulated crystal intensities. The results of this procedure are shown in figure 3. Remarkably, the demodulated crystal intensities closely correspond to those of the incoherent sum of the four compact nominal unit cell transforms shown in figure 2. The resemblance may be quantified by an *R*-factor defined as6.3
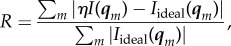
where *η* is a scale factor chosen to minimize *R*. The intensities *I*(***q****_m_*) are the demodulated crystal intensities, *I*_ideal_(***q****_m_*) are equal to the incoherent sum over the four nominal unit cells shown in figure 2. In our case, we find convergence to a value of *R* = 0.07 after 500 patterns. This convergence was largely insensitive to the size and shape of the crystals, but was somewhat slower when the width of the size distribution was increased.

## 7. Phasing intensities from randomly truncated crystals

An approach to phasing symmetry-averaged diffraction data has been described previously by Elser & Millane [28]. Here, we apply a similar approach with a modified intensity constraint appropriate to our problem. Specifically, in our case we assume that the demodulated crystal intensities *I*(***q***) are related to the molecular transform of the asymmetric unit 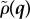 by the approximation7.1
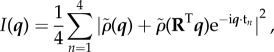
where7.2
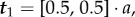
7.3
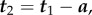
7.4
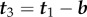
7.5
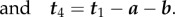
We define two projection operators. The intensity projection 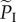 has the action of bringing the magnitudes of the current estimate of the molecular transform, 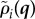, into correspondence with the measured intensities7.6
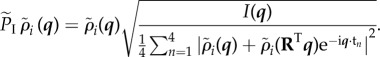


**Figure 4.**
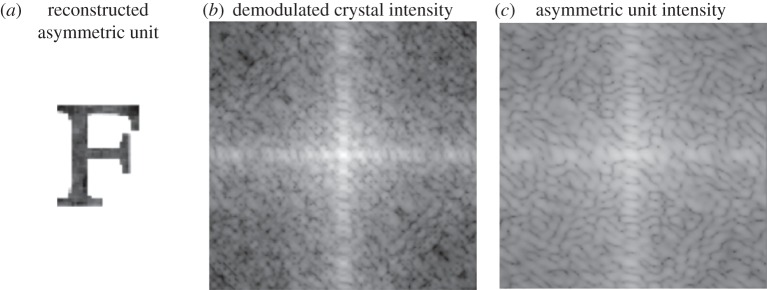
(*a*) A successful reconstruction of the asymmetric unit after 2000 iterations. (*c*) The constrained asymmetric unit intensities were projected onto (*b*) the demodulated crystal intensity constraint according to equation (7.6).

The support projection 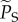 sets the real-space densities to zero in the regions outside of the support *S*7.7
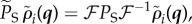
where 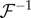 is the inverse Fourier transform and7.8
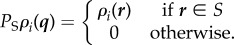
With the two projection operations 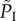 and 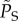, we then apply the difference-map (DM) algorithm (assuming *β* = 1) [29]7.9
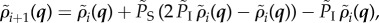
in combination with the error-reduction (ER) algorithm [30]7.10
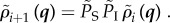
The algorithm switched between the ER and DM algorithms every 10 iterations, beginning with the DM algorithm. The support was updated via the shrinkwrap algorithm [31] every 100 iterations. A successful result, after 2000 iterations in total, is shown in figure 4. The accuracy of this result was quantified by an *R*-factor that compares the demodulated crystal intensities against the average nominal unit cell transform generated from the electron density estimate *ρ_i_*(*r*). Specifically, this *R*-factor was defined as7.11
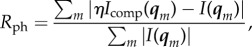
where7.12
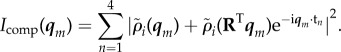
This *R*-factor reduces to a value of *R*_ph_ = 0.07, nearly the same value as *R* mentioned in §6. The near equivalence of *R* and *R*_ph_ suggests that the quality of the reconstruction is limited by the approximate nature of our intensity constraint.

A more rigorous algorithm than the one demonstrated here would ideally use equation (4.7) directly in the intensity constraint, though this would require the introduction of a more sophisticated model for the average sub-lattice cross terms 
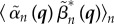
, the parameters of which must be refined in parallel with the phases if the they cannot be determined by other means. Alternatively, it may be possible to better approximate a more general crystal by introducing independent weights on each term of the sum in equation (7.1). These weights must be solved in parallel with the phases as they must be assumed unknown at the first iteration. In our demonstration presented here, we have used the assumption that molecules at the edge of the crystal are randomly truncated, which ensures equal weights in our approximation.

Importantly, we note that there are circumstances where just the integrated Bragg peak intensities are sufficient for unique phasing, particularly for cases in which the solvent fraction (fraction of densities that are uniformly constant) exceeds 50%. Indeed, the simulated crystals shown in figure 3 had a solvent fraction of 62% and can likely be phased without intensities that lie away from the Bragg condition (we include these figures here only for clarity). However, the same algorithm applied to cases where the solvent fractions were 39 and 16% also converged to values of *R*_ph_ = 0.07 or lower, though the greatest fraction of phasing trials succeeded in the cases of higher solvent fractions (about 2/3 at 62%, 1/3 at 39% and 1/10 at 16%). We made no attempt to optimize the algorithm in the cases of higher solvent fractions.

We have not considered the robustness of our algorithm in the presence of measurement errors. Statistical errors in our simulations are induced only by the randomized sampling of crystal sizes and shapes, as well as pixel position jitter, and are smaller than the systematic errors induced by our approximate diffraction model. Assessing the impact of errors should be carried out by implementing the modified intensity constraint within a noise-tolerant phasing algorithm (e.g. [32]), which is beyond the scope of this paper.

## 8. Conclusion

We have raised the point that finite crystals may not have a well-defined physical unit cell. While this realization has no consequence in ‘conventional’ serial femtosecond nanocrystallography, it is of great importance to the development of phasing algorithms that use intensities that are not restricted to the Bragg condition. With some approximation, we have demonstrated that it is possible to determine the structure of an asymmetric unit via iterative phasing techniques for crystals with random edge terminations, and only two molecules per nominal unit cell.

The consequences of our approximations, which neglect interference terms and assume relatively uncorrelated crystal edge terminations, is in need of further consideration. Moreover, the extension to space groups with more than two symmetry-related molecules may give rise to further complications.

## Endnote

1 This expression is correct when the first term of the summation in equation (4.3) is equal to 1. This may always be accomplished by choosing an appropriate origin of the entire crystal, which never affects the diffraction intensity.
